# Sulfur Doping versus Hierarchical Pore Structure: The Dominating Effect on the Fe–N–C Site Density, Activity, and Selectivity in Oxygen Reduction Reaction Electrocatalysis

**DOI:** 10.1021/acsami.1c09659

**Published:** 2021-09-01

**Authors:** Giorgia Daniel, Marco Mazzucato, Riccardo Brandiele, Laura De Lazzari, Denis Badocco, Paolo Pastore, Tomasz Kosmala, Gaetano Granozzi, Christian Durante

**Affiliations:** Department of Chemical Sciences, University of Padova, via Marzolo 1, 35131 Padova, Italy

**Keywords:** PGM-free catalyst, ORR, Fe−N−C, sulfur doping, hierarchical factor

## Abstract

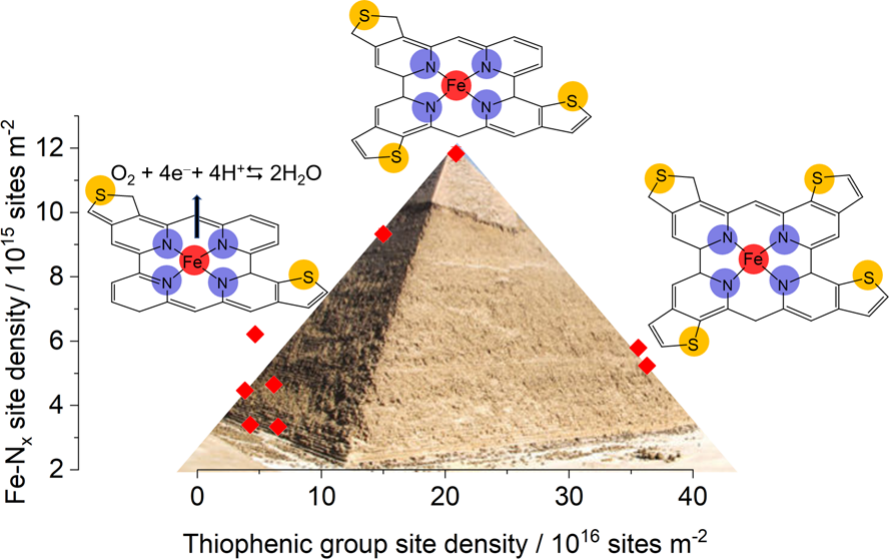

Nitrogen doping has been always regarded as one of the major factors responsible for the increased catalytic activity of Fe–N–C catalysts in the oxygen reduction reaction, and recently, sulfur has emerged as a co-doping element capable of increasing the catalytic activity even more because of electronic effects, which modify the d-band center of the Fe–N–C catalysts or because of its capability to increase the Fe–N*_x_* site density (SD). Herein, we investigate in detail the effect of sulfur doping of carbon support on the Fe–N*_x_* site formation and on the textural properties (micro- and mesopore surface area and volume) in the resulting Fe–N–C catalysts. The Fe–N–C catalysts were prepared from mesoporous carbon with tunable sulfur doping (0–16 wt %), which was achieved by the modulation of the relative amount of sucrose/dibenzothiophene precursors. The carbon with the highest sulfur content was also activated through steam treatment at 800 °C for different durations, which allowed us to modulate the carbon pore volume and surface area (1296–1726 m^2^ g^–1^). The resulting catalysts were tested in O_2_-saturated 0.5 M H_2_SO_4_ electrolyte, and the site density (SD) was determined using the NO-stripping technique. Here, we demonstrate that sulfur doping has a porogenic effect increasing the microporosity of the carbon support, and it also facilitates the nitrogen fixation on the carbon support as well as the formation of Fe–N*_x_* sites. It was found that the Fe–N–C catalytic activity [*E*_1/2_ ranges between 0.609 and 0.731 V vs reversible hydrogen electrode (RHE)] does not directly depend on sulfur content, but rather on the microporous surface and therefore any electronic effect appears not to be determinant as confirmed by X-ray photoemission spectroscopy (XPS). The graph reporting Fe–N*_x_* SD versus sulfur content assumes a volcano-like shape, where the maximum value is obtained for a sulfur/iron ratio close to 18, i.e., a too high or too low sulfur doping has a detrimental effect on Fe–N*_x_* formation. However, it was highlighted that the increase of Fe–N*_x_* SD is a necessary but not sufficient condition for increasing the catalytic activity of the material, unless the textural properties are also optimized, i.e., there must be an optimized hierarchical porosity that facilitates the mass transport to the active sites.

## 1 Introduction

Fuel cell technologies represent an important development for moving to a low-carbon economy, which is expected to offer promising opportunities not only to fight climate change but also to enhance energy security, to revolutionize the transport sector, both for goods and people, and to develop local industries in many countries.^1,2^ Proof of this can be seen in the enormous investments in Europe and in the United States for the development of hydrogen-based technologies, including fuel cells (FC).^3−6^ In fact, even if the large majority of cars still employ fossil fuels, and battery-based electric vehicles are much more diffuse with respect to hybrid hydrogen cars, the FC technology is expected to play a pivotal role in the future for domestic decentralized energy production or in electric vehicles (FCEVs). With further technological improvement, FCEVs will offer very fast refueling time (ca. 3–5 min), greater longevity, better driver experience and safety, and lower cost compared to the actual. Despite the economic and technological problems related to the production, transport, and storage of hydrogen, the main FCs problem is on a different aspect: the high cost due to the low kinetic of the cathode reaction, the oxygen reduction reaction (ORR), and thus, the usage of Pt-based materials as catalysts is still required.^7−9^ With their low cost, high availability, and good tolerance to poisoning, non-precious-metal catalysts (non-PGM) are the best known alternative to Pt.^10−12^ During past decades, various non-PGM catalysts were investigated: M–N–C based on M–N*_x_* sites, non-precious-metal oxide, chalcogenides, and oxynitrides.^13^ The most studied are M–N–C, and among them, the most active metal center is Fe, where iron coordinate from two to five nitrogen functional groups,^14^ and among the different types of Fe–N*_x_* (*x* = 1–5), the metal porphyrin-like Fe–N_4_ site is considered the most important for its ORR selectivity and activity.^15−18^ However, different factors need to be considered to reach good performances, including site density, carbon support hierarchical structure, surface chemistry, graphitization degree, etc.^19−25^ Choosing the right carbon matrix is the turning point to improve catalytic performance; in fact, the increment of the active SD is *per se* not enough to enhance the activity, but it is necessary to rationally design the textural and porous properties of the carbon matrix to facilitate the mass transport between micropores and the bulk solution.^23,26,27^ Moreover, it has been also demonstrated that the incorporation of heteroatoms can influence the catalytic performances.^28^ The idea underlying the doping process is the capability of heteroatoms to modulate the electronic structure of the carbon plane via the delocalization of the π-electrons when pinned into the carbon framework, improving the catalyst activity.^29^ Several studies report that the doping of N atoms improves the ORR activity, which is mainly due to the pyridinic functional group.^30^ In fact, its presence encourages the adsorption of oxygen on the adjacent C atom.^31^ Besides N doping, other heteroatoms (e.g., S, P, and B) can be embedded into N-doped carbon structure generating synergistic effects between the dopants. Considering the S doping, the high electron spin density of doped S atoms enhances the electrocatalytic activity, in particular the asymmetric charge density distribution creates adsorption sites enhancing the performance for the ORR.^32−34^ Another recent study demonstrates that S doping activates carbon atoms next to graphitic N becoming ORR active sites.^35^ However, it is uncharted how the S dopants interact with both Fe-based active sites and N species. The ORR activity of a single Fe–N*_x_* catalytic site seems to be biased by the introduction of electron-withdrawing/-donating groups, so only oxidized S functionality (−SO*_x_*) should induce an increase of the ORR.^36^ However, it has been demonstrated that the main incorporated S structure is thiophene-like (C–S–C) and that its beneficial effect in the ORR was observed to be dependent on the distance between the iron center and the S atom.^37^

The aim of this paper is to understand whether S doping can indeed produce measurable improvements in the formation of Fe–N*_x_* active sites, in the catalytic activity and selectivity of the resulting material, and whether this effect prevails on other material properties such as the hierarchical pore structure or the graphitization degree,^38^ or even if the improvement is due to indirect effect of sulfur on other properties of materials. To do so, here, we employed S-doped mesoporous carbon (SMC) prepared by hard template approach^8,39^ and iron phenanthroline that was used as N and Fe precursor for the formation of Fe–N–C sites.^10,24^

## 2 Experimental Section

### 2.1 Synthesis of the Carbon Supports

Sucrose and dibenzothiophene (DBT) were used as carbon and sulfur precursors, respectively, for the synthesis of different SMCs. Differently doped SMCs were prepared by changing the ratio between the two precursors, and the corresponding mesoporous carbon (MC) have been labeled as SMC (only dibenzothiophene), SMC70 (70:30, dibenzothiophene/sucrose), SMC50, SMC30, and MC (only sucrose). For all of the supports, the preparative procedure consists of the dissolution of 1 g of silica (200 nm particle size, 4 nm pore size, from Sigma-Aldrich) and 1 g of organic precursor in 15 mL of acetone or ethanol depending on the precursor solubility in the medium. H_2_SO_4_ (200–300 μL) was also added to facilitate the oligomerization of the precursors during the impregnation process.^28^ Silica acts as a template matrix, imprinting the mesoporosity to the resulting MCs. The solution was dried in an oven for over 1 h at 100 °C, to remove all traces of solvent until a brownish powder remains. The powder was then treated in a tubular furnace applying a ramp of 5 °C min^–1^ until the temperature reaches 750 °C under N_2_ flow. Once the temperature was reached, the compound was left in the tube for 2 h at a constant temperature. The system was then cooled down until room temperature was reached. The final step for the carbon powder consists of the etching of the template by treating it with liquid solution of 20 mL of NaOH and 20 mL of ethanol in a bath sonicator to ensure the removal of silica from MCs, which was confirmed by the absence of the Si 2p peak attributed to SiO_2_ around 103 eV in the X-ray photoemission spectroscopy (XPS) survey spectrum. The mixture reacts with the silica by dissolving it, while the carbon precipitates and is eventually separated by vacuum filtration on a nylon filter (GVC, nylon 0.2 μm, 47 membrane diameter).^8^

### 2.2 Steam Activation Treatment

The sole SMC sample was subjected to different steam activation treatments. The steam treatment consists of treating at high temperature the carbon powder in a tubular furnace, where the inlet flange lodges a stainless steel needle which tip is fixed at the entrance of the oven.^23^ This apparatus was connected to a syringe pump (SKE Research Equipment). Before performing the activation treatment, the tubular furnace was purged with N_2_, and then the temperature was increased up to 800 °C, and when reached by means of a syringe pump, Milli-Q water at 1 mL min^–1^ was injected with the almost instantaneous water evaporation. The steam atmosphere was maintained for different dwelling times: 5, 20, 40, and 60 min obtaining the samples SMCSt5, SMCSt20, SMCSt40, and SMCSt60, respectively.

### 2.3 Synthesis of Fe–N–C Materials

Iron(II)-phenanthroline chloride (Fe(Phen)_3_Cl_2_) was synthesized from FeCl_2_ and 1,10-phenanthroline monohydrate in ethanol according to literature.^10^ The synthesis of Fe–N–C catalysts was carried out as follow: 200 mg of MCs (SMCX or SMCstX) and 222 mg of Fe(phen)_3_Cl_2_ (2% molar of iron with respect to the molar amount of carbon) were mixed with a ball-miller (Retsch MM 400, 10 mL, and 5 mL steel jars with steel balls) at 20 Hz for 20 min, then heated at 900 °C in a tubular furnace (Carbolite, with a quartz tube ø = 25 mm) for 2 h under nitrogen-hydrogen atmosphere (8% H_2_ in the mixture), and cooled down to room temperature under pure nitrogen flow. The resulting powder was vibro-milled and leached at reflux at 100 °C in 100 mL of 1 M H_2_SO_4_ for 3 h under continuous stirring. The solution was then filtered and washed with at least 500 mL of Milli-Q water using nylon membrane and finally dried in an oven at 80 °C overnight. After the acid washing, the Fe-based powder was heated a second time at 900 °C under H_2_/N_2_ flow as described before. The resulting powders are labeled: FeSMCX, FeMC, and FeSMCStW (where *X* is the percentage of DBT and W is the dwelling time of steam). The final catalysts were ground for 30 min at 24 Hz before the characterizations.

### 2.4 Electrochemical Test

Cyclic voltammetry (CV) and linear sweep voltammetry (LSV) were carried out on a rotating ring-disk electrode (RRDE, Metrohm; *d* = 5 mm GC disk and a Pt ring), in both Ar-purged and O_2_-saturated 0.5 M H_2_SO_4_ solution using an Autolab model 101N potentiostat. All measurements were done in a three-electrode cell thermostated at 25 °C. The RRDE tip was used as the working electrode, a graphite rod was used as the counter electrode, and a homemade RHE as the reference electrode.^40^ RHE consists of a spiral Pt wire settled to the closed end of a capillary glass tube filled with the electrolyte solution in which H_2_ was directly electrogenerated at the Pt wire via the chronoamperometric technique until half of the spiral was filled with gas.

The material activity was investigated on a catalyst layer loaded on GC surface via drop-casting after the preparation of an ink made approximately of an 8:1:1 mixture of water, two organic solvents [chosen from ethanol, acetone, isopropanol, dimethylformamide (DMF), and tetrahydrofuran (THF)], and Nafion (*m*_nafion solution_/*m*_cat_ ≈ 0.8). The dispersion of the powder was ensured using a bath sonicator at a controlled temperature. The loading was chosen to be 0.6 mg cm^–2^ as used in previous works.^11,24^

All of the materials were initially activated in Ar-purged electrolyte with extensive CV cycling at 200 mV s^–1^ until a stable current was observed. In ORR tests, O_2_ was bubbled inside the electrolyte solution for at least 30 min. The number of transferred electron (*n*) was determined by RRDE linear sweep voltammetry according to the following equation
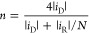
1where *i*_D_ is the current recorded at disk, *i*_R_ is the current recorded at ring, and *N* is the collection efficiency, which is equal to 0.25 (determined by performing RRDE measurement in the presence of K_4_Fe(CN)_6_ in 0.5 M K_2_SO_4_ electrolyte). With the last analysis, it is also possible to evaluate the percentage of hydrogen peroxide produced at the working electrode
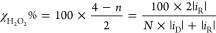
2Other parameters of interest are the half-wave potential (*E*_1/2_), and the limiting current density (*j*_L_) determined from LSV analysis at 1 mV s^–1^ and 1600 rpm. The mass-transport-corrected kinetic current density (*j*_k_) at a selected potential is calculated according to eq 3.
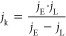
3where *j*_E_ is the current density at the selected potential *E* = 0.8 V vs RHE and *j*_L_ is the limiting current.

To evaluate the catalysts site density, nitrite (NO_2_^–^) poisoning and electrochemical stripping were performed following the procedure described by Malko et al.^41^ This procedure takes advantage of the selective nitrite adsorption on Fe–N*_x_* site, in detail, allowing the calculation of the site density from the charge of NO reductive stripping during a CV measurement. The site density measurements were also performed on a thin layer of catalyst deposited on a GC (RDE, Metrohm *d* = 5.5 mm) in a 0.5 M acetate buffer at pH 5.2; for that reason, the reference electrode was changed to a saturated calomel (SCE). A loading of 0.2 mg cm^–2^ was chosen according to published procedure.

The nitrosyl stripping charge, *Q*_strip_, can be related to the gravimetric site density according to the formula
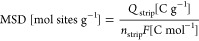
4where *n*_strip_ is the number of electrons associated with the reduction of one adsorbed nitrosyl per site to NH_3_ (or more precisely to NH_4_^+^), which is equal to 5. The turnover frequency (TOF) of Fe–N*_x_* sites is then given by the expression
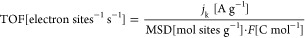
5where *F* is the Faraday constant and *j*_k_ is the kinetic current (or mass activity) determined by the Tafel plot.

### 2.5 Physicochemical Characterization

X-ray photoemission spectroscopy (XPS) measurements were performed at room temperature in a UHV chamber (base pressure <5 × 10^–9^ mbar), equipped with a double-anode X-ray source (Omicron DAR-400) and a hemispherical electron analyzer (Omicron EIS-125). A nonmonochromatized Mg Kα radiation (*h*ν = 1253.6 eV) and pass energies of 50 and 20 eV for the survey and the single spectral windows, respectively, were used. The calibration of the binding energy (BE) scale was carried out using Au 4f^7/2^ as a reference (BE Au 4f^7/2^ = 84.0 eV). The XPS peak of nitrogen was deconvoluted into single components using symmetrical Voigt functions.

Raman spectra were recorded using a micro-Raman setup with a 0.7 mW laser at 633 nm with 20× LWD objective (pinhole 25 μm). N_2_ adsorption/desorption isotherms were recorded at 77.3 K using an ASAP 2020 Plus instrument. Specific surface area of the samples was determined by the Brunauer–Emmett–Teller (BET) analysis and with the quenched-solid density functional theory (QSDFT) model. It takes into account the interconnectivity among pores, the interactions between adsorbent and adsorbate, and the roughness of porous surface. The total volume of pore was obtained applying Gurvitsch law at *p*/*p*^0^ ≈ 0.98. Elemental analysis (EA) was carried out using a Thermo Scientific Flash 2000 analyzer. Transmission electron microscopy (TEM) images were obtained with an FEI TECNAI G2 instrument operating at 100kV.

An Agilent Technologies 7700× inductively coupled plasma-mass spectrometer (ICP-MS) was employed for inductively coupled plasma-mass spectrometry analysis. The samples (10 mg) for ICP analysis were treated for 1 h at 100 °C using 2 mL of concentrated nitric acid immersed in a water bath.

## 3 Results and Discussion

**Figure 1:**
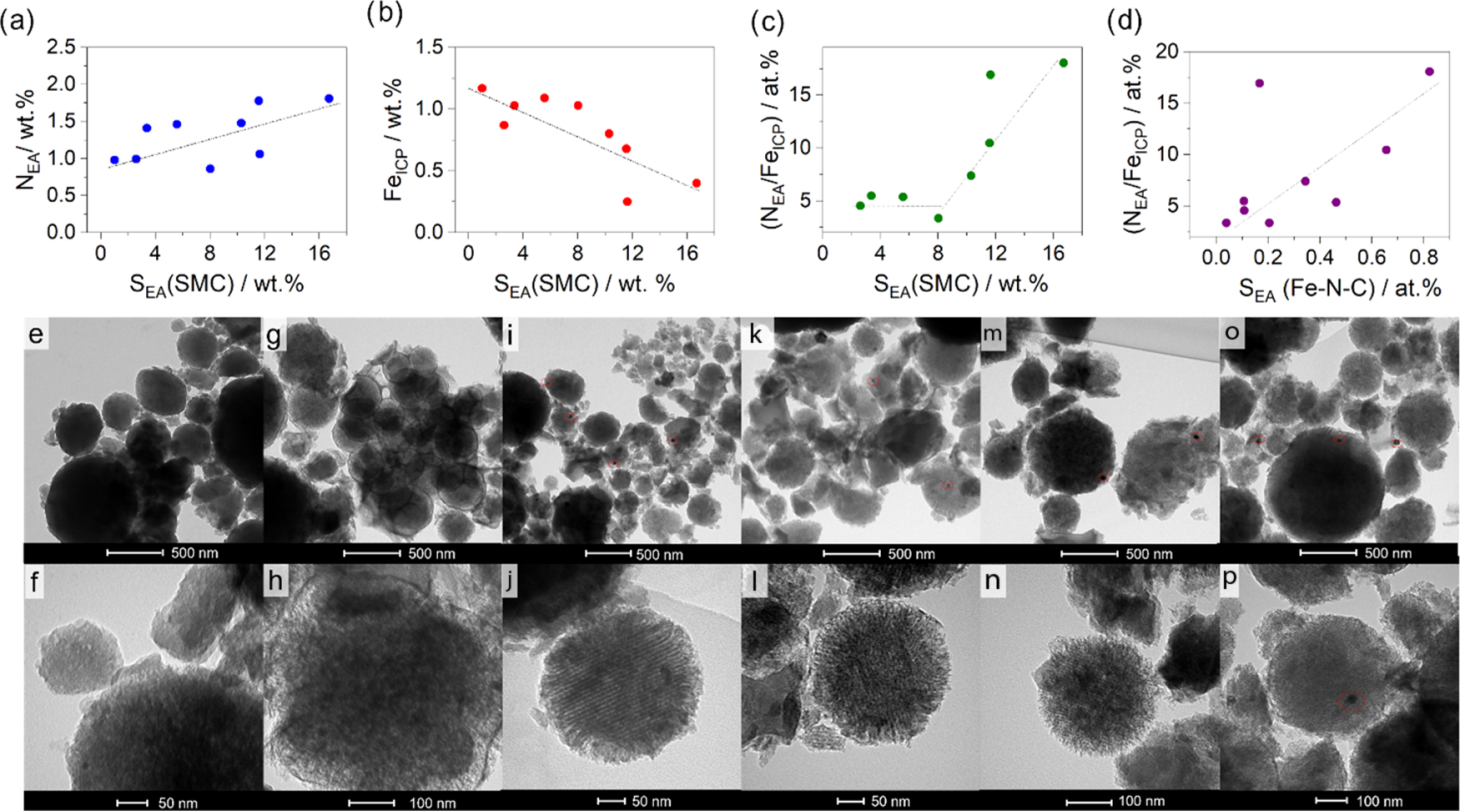
(a–d) Correlation of element quantity determined from EA or ICP-MS. SMC and Fe–N–C are meant to identify the carbon supports and the catalysts, respectively (dotted lines are only intended to guide the eye); (e–p) TEM images of (e, f) FeSMC, (g, h) FeMC, (i, j) FeSMCSt5, (k, l) FeSMCSt20, (m, n) FeSMCSt40, and (o, p) FeSMCSt60. Red circles indicate the presence of iron NPs.

The S-doped supports were synthesized using a hard template approach; the reader may refer to the Experimental Section for details. The P200 is a commercial mesoporous silica composed of spherical particle of 200 nm in diameter, with inner pore size of 4 nm and a declared surface area of 823 m^2^ g^–1^.^8^ The hard template synthesis allows us to synthesize MC with superimposable morphology (same particle dimension and shape), while the chemical functionalization can be varied at will (different content of sulfur functional groups). The steam activation at 800 °C of SMC at different dwelling times in the oven chamber was also evaluated with the aim of understanding the effect of textural properties on Fe–N*_x_* sites formation. In fact, it is expected the steam treatment can improve the textural properties (pore surface area and volume) of the carbon supports and the relative content of graphitized carbon. The obtained carbon supports were employed for the preparation of Fe–N–C catalysts by the thermochemical reaction of a mixture of the modified carbon support and Fe(Phen)_3_Cl_2_. The thermal treatments can generate small etching molecules such as SO_2_, CO, and CO_2_ that can induce a modification of the textural properties as previously observed also with other doped carbon supports.^8,42^ CHNS elemental analysis confirmed the successful doping of the SMC samples (Table 1), which have sulfur contents proportional to the dibenzothiophene/sucrose ratio employed in the synthesis. When 100% DBT was used, the sulfur percentage reaches 16.71%, and it decreased to 11.57, 10.29, and 5.57% as the DBT/sucrose ratio is decreased to 70:30, 50:50, and 30:70, respectively. The sample prepared by the sole sucrose, as expected, does not show appreciable sulfur (Table 1, entry 5). The steam treatment performed on SMC has the effect to decrease the sulfur content and the residual sulfur is proportional to the treatment time, i.e., the more the sample is exposed to the action of the steam at high temperatures, the more the sulfur content decreases. In fact, the sulfur content decreases from 16.71 to 2.60% passing from 0 to 60 min of treatment (Table 1). This can be explained by the fact that the steam tends to react with the amorphous component of the sample and with graphitic grain boundaries, where the sulfur functional groups are located.^43,44^ In Fe–N–C catalysts sulfur functional groups are still present but in a different percentage with respect to the pristine carbon supports (Table 1). In fact, the percentage of sulfur remains proportional to the initial content, i.e., FeSMC shows the highest percentage (2.35%) while the lowest percentage is observed in FeMC (0.11%), and intermediate values are observed for Fe–N–C samples prepared from carbons with a decreasing ratio of dibenzothiophene to sucrose (Table 1). The set of Fe–N–C catalysts prepared from steam-activated SMC show even lower sulfur values in the range 0.6–0.3 wt %. It is interesting to note that the nitrogen percentage in FeSMC samples shows a certain proportionality with the sulfur content initially present in the carbonaceous media, i.e., the nitrogen content is higher where the sulfur content was also initially higher (Figure 1a). Even if the data are scattered and a perfect linear correlation cannot be obtained, the trend is clear. An opposite trend can be observed for iron determined via ICP-MS (the Fe content spans between 0.25 and 1.17 wt %), i.e., the iron content remaining after heat treatments and acid washing scales inversely to the initial sulfur content (Figure 1b). Considering that there must be some proportionality between iron and nitrogen content, as the ORR active sites consist of a metal center capable of coordinating two to five nitrogen atoms, Figure 1c shows the N/Fe ratio against the sulfur content in the carbonaceous support, expressed as atomic percentage. It is worth reminding that Fe contents estimated by ICP cannot represent the Fe–N*_x_* contents, but it gives an overestimation of Fe–N*_x_* sites, since the presence of iron nanoparticles (NPs) leads to the concomitant presence of iron oxides, although these remain inactive due to a carbonaceous coating that does not allow their dissolution during the acid treatment.^11^ However, the iron content not in the form Fe–N*_x_* can be treated as a systematic error and therefore even if not indicative of absolute value it, becomes so in relative value. What can be observed is that ideal N/Fe ratio to form Fe–N_4_ can be easily obtained even at a low sulfur percentage, but the fixation of nitrogen in carbonaceous material occurs more readily than that of iron, when the sulfur content increases markedly. This is why a step-like trend is observed instead of a simple linear trend. If we now consider the N/Fe ratio in relation to the sulfur content remaining in the carbonaceous support, after Fe and nitrogen fixation, we can see that there is a certain proportionality Figure 1d, and this is a further confirmation that the presence of sulfur in the support drives the formation of nitrogen (and possibly Fe–N*_x_* surface functional groups), as will be seen more specifically later in the text. However, on the sole basis of stoichiometry considerations, Figure 1c,d points out that, with iron being the limiting element, most part of nitrogen functional groups are not involved in the Fe–N_4_ site, but in other sites that however are known to have an effect on ORR electrocatalysis.^45^

**Table 1:** Bulk Composition Derive from Elemental Analysis and ICP-MS (Fe) Analysis

	C_support_^a^ (wt %)	S_support_^a^ (wt %)	C_cat_^a^ (wt %)	H_cat_^a^ (wt %)	N_cat_^a^ (wt %)	S_cat_^a^ (wt %)	Fe_cat_^b^ (wt %)
FeSMC	75.18	16.71	78.37	1.20	1.81	2.35	0.40
FeSMC70	57.20	11.57	67.54	1.11	1.78	1.81	0.68
FeSMC50	75.82	10.29	82.47	0.66	1.48	0.94	0.80
FeSMC30	61.87	5.57	85.34	0.45	1.46	1.24	1.09
FeMC	90.55	<1	75.18	0.99	0.98	0.11	1.17
FeSMCSt5	78.44	11.64	79.41	1.17	1.06	0.48	0.25
FeSMCSt20	85.63	8.02	78.57	1.07	0.86	0.58	1.03
FeSMCSt40	68.40	3.36	76.07	1.28	1.41	0.31	1.03
FeSMCSt60	83.75	2.60	79.46	1.13	0.99	0.31	0.87

a Determined from CHNS elemental analysis.

b From ICP-MC analysis.

The morphology of the S-doped supports was evaluated through TEM images (Figures 1 and S1). Figure S1a–c shows an array of SMC spheres, with a mean diameter of ∼250 nm and characterized by an internal porous structure conforming to the silica template. Focusing on the border of one sphere, the pore channels can be recognized (Figure S1b). The SMC materials doped with different S amount are very similar in shape and pore structure, attesting the solidity of the adopted hard template synthesis. The steam effect on morphology was also investigated (Figure S1d–f). The spherical shape was maintained even though the carbon particles appear less dense, and in some spheres, the porosity channels disappear or become less ordered, while in other spheres, edges become more fragmented. Therefore, the action of the steam activation is to open up the pore structure, increasing the hierarchy to the detriment of the pore order.

Figure 1 shows TEM images of some Fe–N–C catalysts: the morphology of the support is maintained after the Fe/N doping and the pyrolysis treatment and it is evident how the carbon particle dimensions span from hundreds to thousands of nanometers. In fact, the pore channels in Figure 1e of FeSMC are evident, only the sphere borders are jagged and not defined, maybe due to the carbonization of phenanthroline used as a ligand in the iron complex. The spherical structure of FeMC appears quite damaged (Figure 1g,h), and some porous carbon sheets are also visible even if not being present in the starting MC. In FeSMCSt*X* (*X* = 5, 20, 40) two main structures are visible: one characterized by ordered pores and the other one by disordered pores (Figure 1i–r). Similar structures, even if less ordered, were also observed in FeSMCSt60 (Figure 1q,r). It is worth mentioning that the border of carbon particle not only became more jagged, but the edge erosion extension grows for longer treatment. Very few iron nanoparticles (NPs) are individuated embedded in the carbon structure, and this is reasonable considering the low iron content reached after the acid wash step (Table 1 and Figure 1i–r).

**Figure 2:**
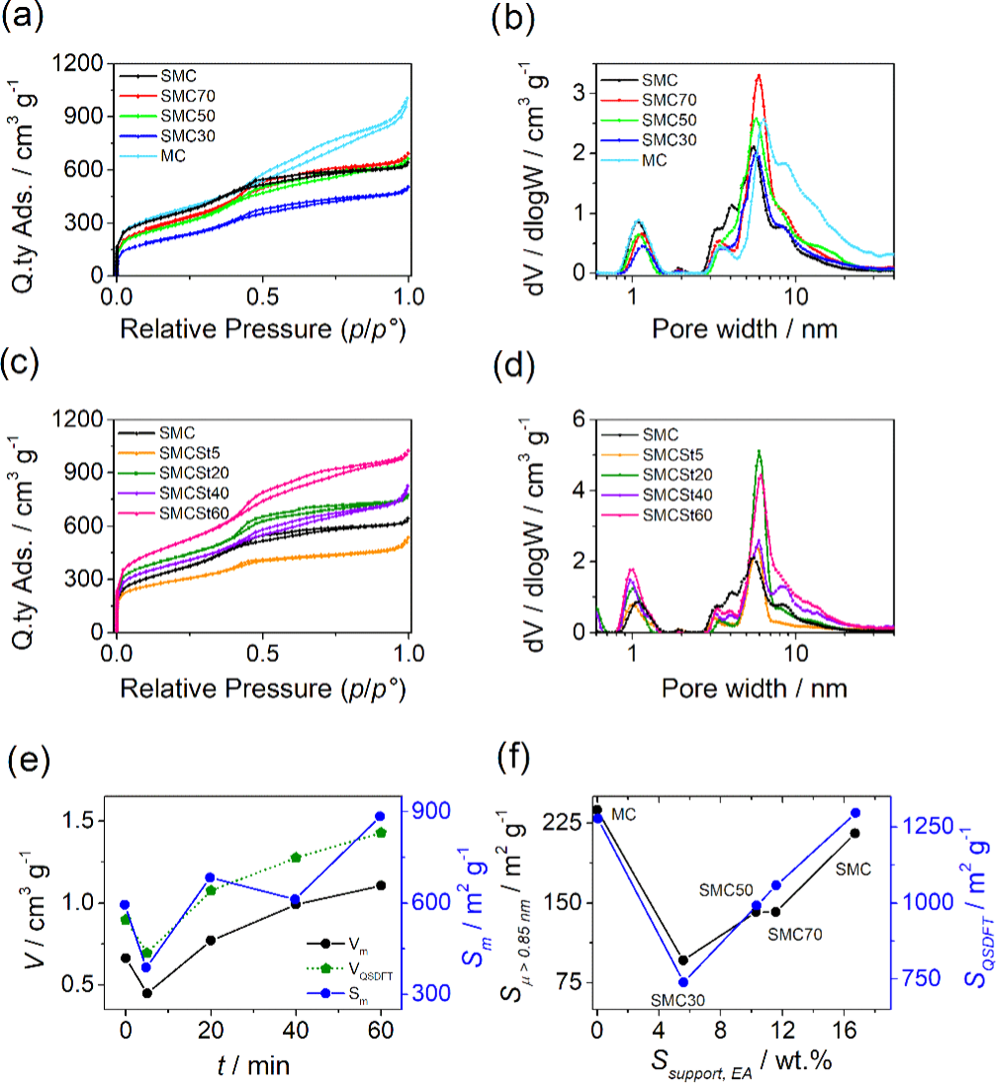
(a) N_2_ adsorption/desorption isotherms and (b) pore size distribution (PSD) of SMC series with different contents of sulfur. (c) N_2_ adsorption/desorption isotherms and (d) pore size distribution of SMCSt series. Correlation of textural properties (e) with the steam treatment duration and (f) with the amount of sulfur fixed in the precursors.

The textural properties, which consider another grade of morphology characterization, were evaluated using N_2_ adsorption/desorption isotherm, and the data are resumed in Table 2. The isotherms in Figure 2a belong to IV(a) type with an H4 hysteresis. The adsorption branch follows a hybrid-type I(b)/II isotherm, which presents two main features: (i) a pronounced uptake at a low relative pressure (*p*/*p*^o^) associated with the micropore filling and (ii) a slow gradual increment of N_2_ adsorbed up to 0.8 *p*/*p*^o^.^46^ These effects are typical of a micro-mesoporous material.

**Table 2:** Summary of Various Textural Parameters Derived from Isotherm Analysis

	***S***_QSDFT_ (m^2^ g^–1^)	***S***_μ_ (m^2^ g^–1^)	***S***_m_ (m^2^ g^–1^)	***V***_QSDFT_ (cm^3^ g^–1^)	***V***_μ_ (cm^3^ g^–1^)	***V***_m_ (cm^3^ g^–1^)	***V***_tot_ (cm^3^ g^–1^)
SMC	1296	701.7	594.2	0.898	0.235	0.662	0.995
SMC70	1058	373.8	684.2	0.951	0.135	0.816	1.071
SMC50	992	344.9	646.7	0.912	0.126	0.786	1.026
SMC30	739	243.5	495.7	0.698	0.090	0.608	0.779
MC	1277	558.3	718.7	1.304	0.209	1.095	1.554
SMCSt5	1191	803.5	387.6	0.695	0.247	0.448	0.831
SMCSt20	1562	878.6	683.2	1.075	0.305	0.770	1.197
SMCSt40	1377	764.5	612.1	1.276	0.286	0.990	1.277
SMCSt60	1726	841.0	884.9	1.429	0.323	1.106	1.585

The sulfur content in the SMCX series seems to affect the specific surface area (*S*_QSDFT_): i.e., the higher the sulfur, the higher the microporous surface area (*S*_μ_). The sample obtained from sucrose alone (MC) has a surface area close to the support obtained from pure DBT (SMC), but the micro- and mesoporous surfaces ratio is different. In fact, MC has a lower *S*_μ_ and a higher *S*_m_ compared to SMC, which slightly changes the respective isotherms up to 0.5 *p*/*p*^o^, i.e., the adsorption branch at 0.5 *p*/*p*^o^ increases faster in MC than in SMC. From 0.5 onward, the evolution of hysteresis and gas adsorption is indeed different: SMC has an H4 hysteresis, where the desorption branch approaches to the adsorption one at 0.45 *p*/*p*^o^ (the characteristic cavitation pressure), while MC features a mixed hysteresis between H3 and H4 without a sharp step-down of the desorption resulting in a higher total pore volume. This difference is due to the porous structure of the supports: SMC has open and restricted pores, instead MC has a more open and accessible surface. The isotherms of the other supports (SMC70, SMC50, and SMC30) are more similar to SMC than to MC. To obtain the porous parameters, namely, the pore size distribution (PSD), a slit/cylindrical/spherical QSDFT adsorption model was applied on experimental data,^47−49^ and it was chosen considering both the hysteresis features and the structure of the P200 silica template. Comparing the pore size distribution between SMC and MC (Figure 2b), it is possible to observe that they have similar micropore size peaked at 1.09 nm, while they differ for the size of mesopore. MC has a shoulder toward higher values compared to SMC with two peaks at 6.4 and 8.5 nm. Reducing the content of DBT precursors, the microporous volume is reduced, and the width increases slightly up to 1.15 nm, while the mesopores are not affected.

The effect of steam on textural properties was also evaluated. The shape of the isotherm is not affected by steam and so the type (IV(a) type) remains the same, while the hysteresis shape change (Figure 2c). In general, steam opens the structure acting on the mesopores, which are centered around 6 nm as shown in Figure 2d. The microporous surface area lies between 804 and 879 m^2^ g^–1^, while the mesoporous area is increased from 594 to 885 m^2^ g^–1^ for SMC and SMCSt60, respectively. SMCSt5 does not present an increment of surface area and its pore size distribution is less broad with pores of 1 and 6 nm. In this case, steam does not affect the texture, but it rather removes some amorphous carbon, as it will be shown later on in the text when discussing the Raman spectrum. SMCSt20 hysteresis is characterized by two step-downs in the desorption branch at 0.6 and 0.4 *p*/*p*^o^, which could identify two types of ink-bottle shape pores.^46,50,51^ Increasing the treatment time, in SMCSt60, the first step-down at 0.6 *p*/*p*^o^ becomes less evident according to the enlarging of pore size from 7 to 20 nm, as demonstrated by pore size distribution in Figure 2d. SMCSt40 lies out of trend with a decrease of *S*_QSDFT_ and *V*_μ_, but a clear explanation cannot be given about this.

Figure 2e reports some textural properties as a function of the steam treatment duration, and it is evident that for St = 5 min, there is a diminution of the mesopore surface and volume that was explained considering a possible clogging of the pore structure. However, for more extensive treatments, *V*_m_ and *S*_m_ increase with increasing treatment duration, with the sole exception of the point at St = 40 min, for which there is no easily interpretable decrease in the sole mesopore surface.

Figure 2f shows that as the content of sulfur, and therefore of DBT, increases, there is an almost linear increase in both *S*_QSDFT_ and *S*_μ_. It is interesting to note that the sample prepared from sucrose alone has comparable *S*_QSDFT_ and *S*_μ_ to those of SMC, and therefore it is the different ratio of the two precursors that enables the tuning of micropore size (here greater than 0.85 nm). Therefore, sulfur has a dual effect: (i) it is able to dope the carbonaceous material and (ii) it has a porogenic action.^8^

**Figure 3:**
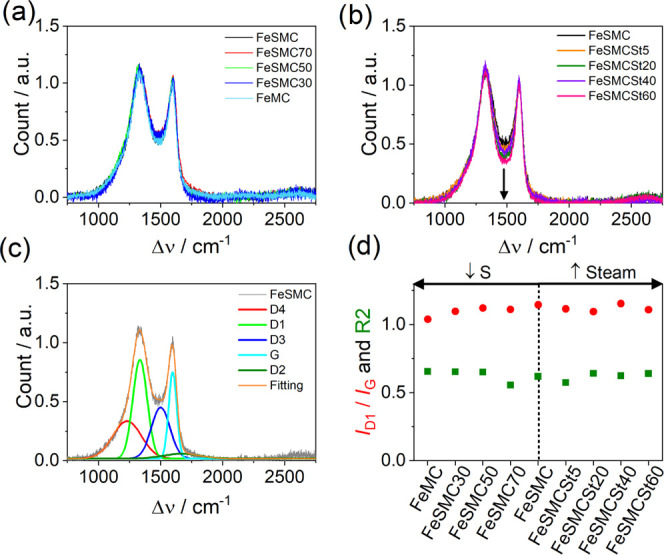
(a, b) Comparison of Raman spectra for the two sets of samples, (c) deconvolution of Raman spectrum using gaussian peak functions for FeSMC catalyst, and (d) *I*_D1_/*I*_G_ ratio and *R*_2_ parameters for the different catalysts.

The graphitization degree was evaluated on the Fe–N–C catalysts by means of Raman analysis. These materials do not present defined second-order Raman spectra (2400–2700 cm^–1^ region), and only a smeared flat band is present, which overlaps with the background noise (Figure 3a,b). It is clear that even if the sulfur content significantly differs among the FeSMCX samples, this does not lead to substantial changes, as confirmed by the superimposable Raman spectra reported in Figure 3a. The spectra deconvolution in the first-order region between 1100 and 1700 cm^–1^ (Figure 3c) was performed using five gaussian bands: *D*_4_, *D*_1_, *D*_3_, *G*, and *D*_2_, with the latter fixed at 1620 cm^–1^, as reported by Sadezky et al.^52^ The position of the main bands D1 and G did not significantly change passing from FeSMC to FeMC (Table S1). The *A*_D1_/*A*_G_ and *I*_D1_/*I*_G_ ratios are close to 2.0 and 1.1, respectively, and that is an indication of carbon material with a poor ordered structure (Figure 3d).^53^ A further confirmation of this is the parameter *R*_2_, which for poorly organized carbon materials assumes a value >0.5, while *R*_2_ < 0.5 is indicative of highly graphitized carbons, where *R*_2_ = *D*_1_/(*G* + *D*_1_ + *D*_2_) is the ratio between the area of *D*_1_ peak and the sum of *G*, *D*_1_ and *D*_2_ areas (Table S1). In the present case, *R*_2_ remains essentially constant passing from samples with higher to low sulfur content.^54^ The high *R*_2_ and *I*_D1_/*I*_G_ ratio are to ascribe to sulfur, which has a larger atomic radius than carbon so that the incorporation of sulfur induces more strain and defect sites in the carbon matrix, thus resulting in a lower graphitization degree, and to smaller carbon crystallite dimensions.^55,56^ A slightly different picture emerges from the FeSMCSt series where the steam treatment leads to a 13% reduction of the D3 band area, which accounts for amorphous carbon (Figure 3b and Table S1). However, the *I*_D1_/*I*_G_ ratio still remains high, stabilizing around 1.1 (*A*_D1_/*A*_G_ around 2.1), and *R*_2_ stems between 0.55 and 0.65. Both values are typical for a low-ordered carbon structure (Figure 3d).^57^ Therefore, it may be inferred that all of the catalysts possess similar properties in terms of graphitization degree (small carbon crystallites dispersed in amorphous carbon), but they differ in terms of sulfur content and textural properties.

**Figure 4:**
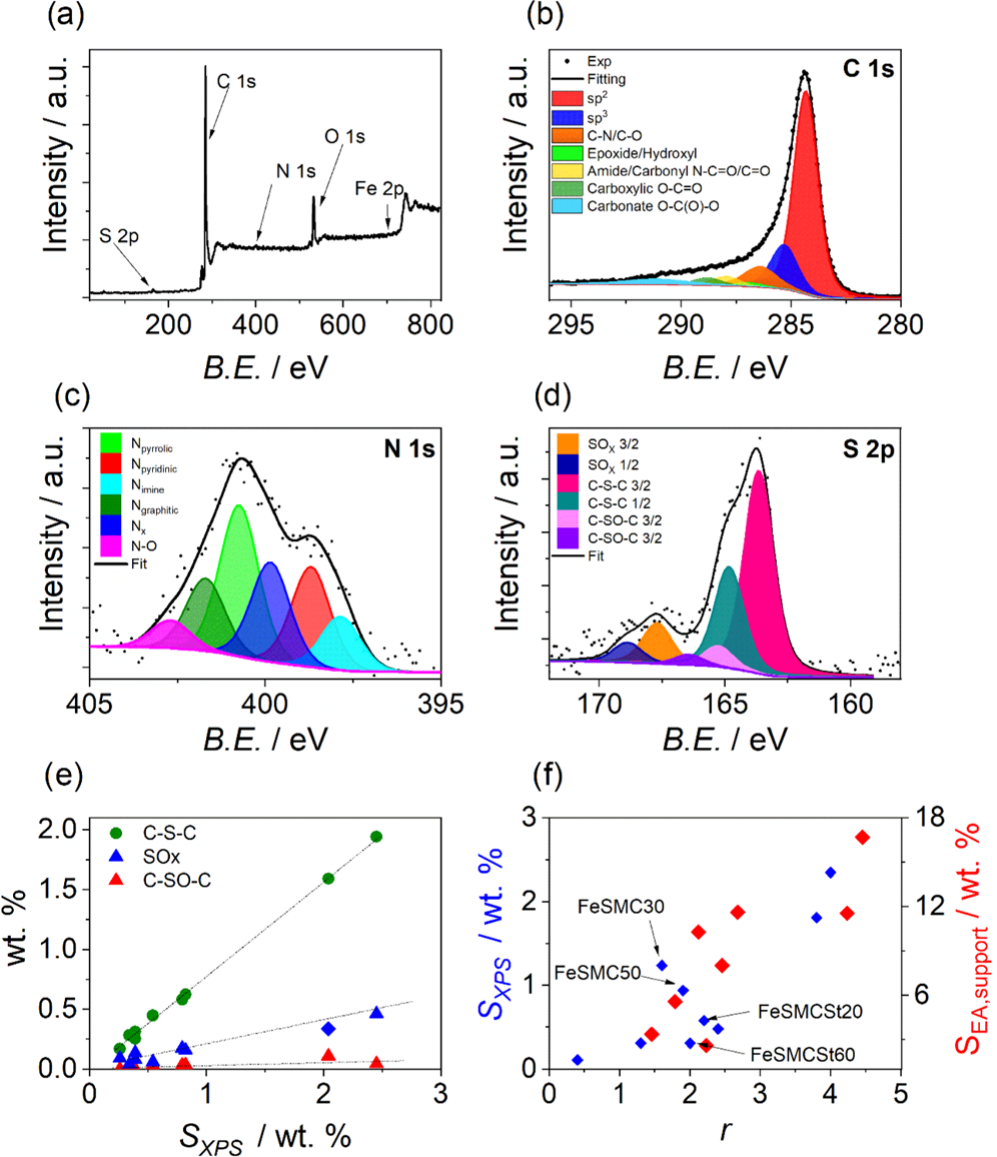
(a) Survey of FeSMC. Examples of high-resolution core-level XPS spectra of (b) C 1s, (c) N 1s and (d) S 2p of FeSMC. (e) Variation of different sulfur components (C–S–C, SO*_x_*, and C–SO–C) with the total sulfur content determined by XPS measurements and S 2p deconvolution. (f) Variation of *r* parameter with the sulfur content determined by elemental analysis (red marks) and XPS (blue marks); *r* parameter is calculated according to equation *r* = (C–S–C)/Fe (atom %) and accounts for the ratio between the thiophenic functional groups and iron content.

Catalyst surface compositions were determined by XPS; an example of XPS survey, N 1s, C 1s, and S 2p are reported in Figure 4, whereas data of speciation are resumed in Table S2. XPS analysis confirmed that a high sulfur content in the initial support allows us to fix a higher percentage of nitrogen in the carbon surface, the opposite thing happens for iron, which actually decreases in content with the increase of the initial sulfur content, as already pointed out in the previous paragraph.

Figure 4b shows the C 1s spectra of analyzed samples. The C 1s spectra were fitted according to refs (23, 58−61). The main carbon component at BE of 284.3–284.4 eV is attributed to C sp^2^. At higher BE (285.4 eV), the peak represents the sp^3^ C–C bond. There is also a substantial amount of carbon bound to nitrogen or/and to oxygen (peak at 286.4 eV). Peaks at BEs higher than 286.4 eV are due to carbon species bound to oxygen (C=O, O–C=O, O–C(O)–O) or oxygen and nitrogen (C–N=O, N–C=O). The peak at 291.1 eV is due to shakeup satellite (π–π*). In the literature, the position of the C–S–C bond ranges from 285.0 to 286.0 eV,^62−66^ moreover taking into account that samples with Fe and S contain less than 1 atom % of sulfur, which would account for less than 2 atom % of fraction of C 1s peak (assuming that one S is bounded to two C). Therefore, the assignment of the C–S–C position in the analyzed sample is not trivial. In the literature,^62^ in a sample containing ca. 40% of C–S–C, the C 1s position assigned to C–S–C is at 285.3 eV, while sp^2^ C is at 284.3 eV; therefore, it can be assumed that the C–S–C peak overlaps with the C sp^3^ component.

The surface content of nitrogen determined by the XPS analysis well matches with the bulk content determined by the elemental analysis, confirming a homogeneous functionalization of the carbonaceous substrate.^53^ The N 1s high-resolution signal was fitted in several components including:^67^*N*-imine (397.8 eV), *N*-pyridinic (398.8 eV), *N*-amine and N*_x_* (399.9 eV), *N*-pyrrolic (400.7 eV), *N*-graphitic (401.7 eV), and *N*-oxide (402.7 eV) (Figure 4c). Pyrrolic nitrogen is distinguished among all of the other functional groups because it is the most abundant. Conversely, the N*_x_*-Fe functional group, which is supposed to be the most important active site for ORR, reaches its highest value (25.1 wt %) in FeSMCSt40. However, this result must be taken with caution since N*_x_*-Fe and *N*-amine signals fall at similar BE, making XPS analysis not fully reliable in quantifying N*_x_*-Fe active sites, that is why sometimes only one component is used. The S 2p signal for sulfur is deconvoluted into three species: S-thiophene (C–S–C) at 163.6 eV,^68^ sulfoxide (C–SO–C) at 165.3 eV, and S-oxide (oxidized S or SO*_x_*) at 167.6 eV (Figure 4d).^69^ Each peak of sulfur-based functionalities is split into two spin–orbit components, namely, 3/2 and 1/2. The percentage of the different species are reported in Table S2. It is proposed in the literature that the catalytic activity of the Fe–N*_x_* site can be tuned introducing electron-withdrawing/-donating groups close to the active site, but which of the two effects actually determines the positive influence on the activity of the Fe–N*_x_* site toward the ORR is the subject of conflicting and even antithetical opinions.^36,70^ In fact, Mun et al. explained using DFT and XANES analysis that in catalysts with a high ratio of oxidized S (SO*_x_*)/(C–S–C), there is an electron-withdrawing effect that removes charge density from Fe–N*_x_*, decreasing the d-band center so that the intermediate adsorption energy becomes lower, thereby facilitating the ORR.^36,71^ Conversely, Ni et al. found that thiophene functionality donates electron density to the carbon plane thanks to its lone pair, and this could tune Fe–N_4_ activity for the ORR.^72^ Therefore, the activity of Fe–N*_x_* sites could be controlled by changing the extent of the electronic effect.^36^ Moreover, the position of the sulfur is important, and it is beneficial if located at least 7.3 Å away from iron active site. Figure 4e shows that the percentage of thiophene-like sites increases as the whole sulfur content in the carbonaceous support increases, as does the SO*_x_* group, even though the latter is always present in much lower percentages. Ni et al. also suggest that the relative ratio of sulfur and iron is crucial to the coordination configurations of the active sites, which directly determine the ORR activity. To do so, they introduced a parameter *r* = (C–S–C)/Fe (atom %) that accounts for the ratio between the thiophenic functional groups and iron content, and found an optimal ratio of 1.81, i.e., the higher catalytic activity in terms of *E*_1/2_ was found in those catalysts where there were almost two thiophenic groups per iron atom. It is worth noting that *r* increases with increasing sulfur content in the carbon support (Figure 4f) and in particular from 0.4 to 4.3 passing from FeMC to FeSMC (Table S2), i.e., from the support with the lowest amount of sulfur to the one with the highest amount, where FeSMC50 assumes almost the optimal value (*r* = 1.9), and therefore, it is expected to perform better than the other catalysts. On the other hand, the samples with excess sulfur content are expected to not perform properly since the formation of FeS*_x_* species, which according to the literature are removed after acid washing, competes with Fe–N_*x*_ formation.^72^ Finally, looking to the survey (Figure 4a), no evident peak of Silicon was observed, confirming the effectiveness of the carbon etching process.

**Figure 5:**
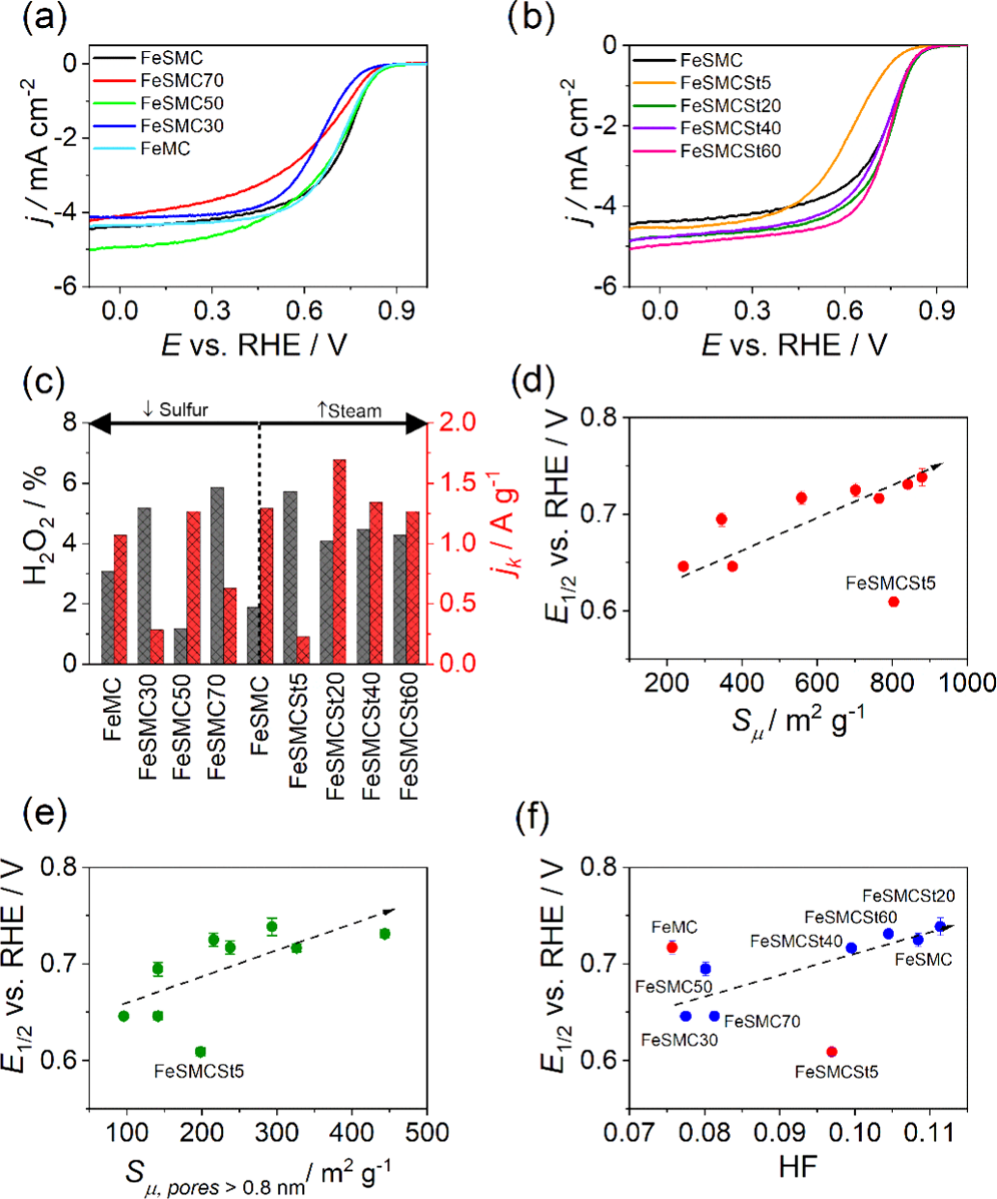
(a, b) LSV recorded at 1 mV s^–1^ in O_2_-saturated electrolyte (0.5 M H_2_SO_4_) at 1600 rpm; (c) kinetic current density determined at 0.8 V vs RHE and H_2_O_2_ generation determined at 0.2 V vs RHE for different catalysts. Variation of the half-wave potential with different textural properties: (d) surface area of micropore, (e) surface area of micropore bigger than 0.8 nm, and (f) hierarchical factor (HF). In (d–f), dotted lines are only intended to guide the eye.

Cyclic voltammetry and linear sweep voltammetry at RRDE were employed to evaluate the effect of different sulfur content and textural properties on the catalytic activity of the different catalysts (Figure 5). It is worth noting that FeSMC and FeMC have almost superimposable electrochemical behavior with very similar *E*_1/2_ and limiting current *j*_L_ for O_2_ reduction process. FeSMC50 shows a similar electrochemical performance even if a higher *j*_L_ is reached (Figure 5a). FeSMC, FeSMC50, and FeMC have also similar *j*_k_ at 0.85 V vs RHE, and the number of transferred electrons (*n*) is close to 4 with ca. 6% of maximum peroxide production (% H_2_O_2_ in Figure 5c). In the same series, FeSMC30 and FeSMC70 have definitively lower performance (Figure 5a) in terms of *E*_1/2_ and *j*_k_ and the selectivity is more or less similar (Table S3). So, there appears to be no direct dependence between *E*_1/2_ or *j*_k_ and the sulfur content determined by elemental analysis or via XPS in the final catalyst. Even the speciation into (C–S–C) or SO*_x_* groups does not allow for any correlation, and this is evident since both defects increase in content as the total sulfur content increases, as seen in the previous paragraph. Not even the volcano-type dependence of *E*_1/2_ from (C–S–C)/Fe ratio proposed by Ni et al. finds confirmation in the sulfur-doped series.^72^

The samples treated with steam show, with the exception of FeSMCSt5, the same behavior in the kinetic-controlled zone, while they differ in the diffusive-controlled zone by a different limiting current, i.e., the sample with a prolonged steam treatment shows a higher limiting current (Figure 5b). This can be associated with the opening of the pore structure, which results in a better utilization of the active sites and allows the FeSMCSt60 sample to become one of the best catalysts, despite a sulfur content that decreases dramatically after steam activation (Figure 5c and Table S3). The exceptional behavior of FeSMCSt5 can be explained considering that such brief steam treatment does not allow an effective pore opening but the sole partial oxidation of the most reactive amorphous carbon, and this is confirmed by the reduction of mesoporous volume from 0.662 to 0.448 cm^3^ g^–1^.^26^ Thus, at first sight, sulfur does not appear to have a univocal effect on the catalytic properties of the material, but it is worth checking whether or not the textural properties, which depend, as we have seen, on the sulfur content, can have an effect on the performance of the material. In fact, textural properties are well known to contribute to the final activity, determining the exposure of active sites and consequently the effectiveness of mass transport.^23^Figure 5d shows that there is a good correlation between the half-wave potential and the micropore surface area, i.e., *E*_1/2_ becomes more positive (the catalytic activity increases) when the micropore surface area increases. It is known that micropore surface is fundamental to increase the activity because they are involved in the formation of Fe–N*_x_* centers.^73^ FeSMCSt5 does not fit in the trend, but if the sole pore larger than 0.8 nm is considered, there seems to be a better congruence with the other catalysts (Figure 5e). Furthermore, *E*_1/2_ shows a good correlation also with the hierarchical factor (HF) (Figure 5f), which is a descriptor of how much interconnected are micropores and mesopores. HF is expressed by the equation
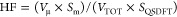
6where *V*_μ_ and *V*_TOT_ are the volume of micropore and the total volume pore, respectively, while *S*_m_ and *S*_QSDFT_ are the surface area of mesopore and the total surface area determined by the quenched-solid density functional theory model, respectively.^24^ In the best catalysts, the HF factor is maximized by increasing the surface area of the mesopores without extremely decreasing the volume of the micropores, with the aim of improving the efficiency of reagent and product transport to the active site. In the present case, the catalysts showing a maximized HF are those that showed improved catalytic activity, such as FeSMC or FeSMCSt20, while the worst catalysts are those with the lowest HF (Figure 5f), although the sulfur content of the carbon support or remaining in the catalyst may be higher than in other, better-performing catalysts (Tables 1 and S2). Therefore, not only microstructure is important, but also mesostructure is useful to obtain an active catalyst toward the ORR.

**Figure 6:**
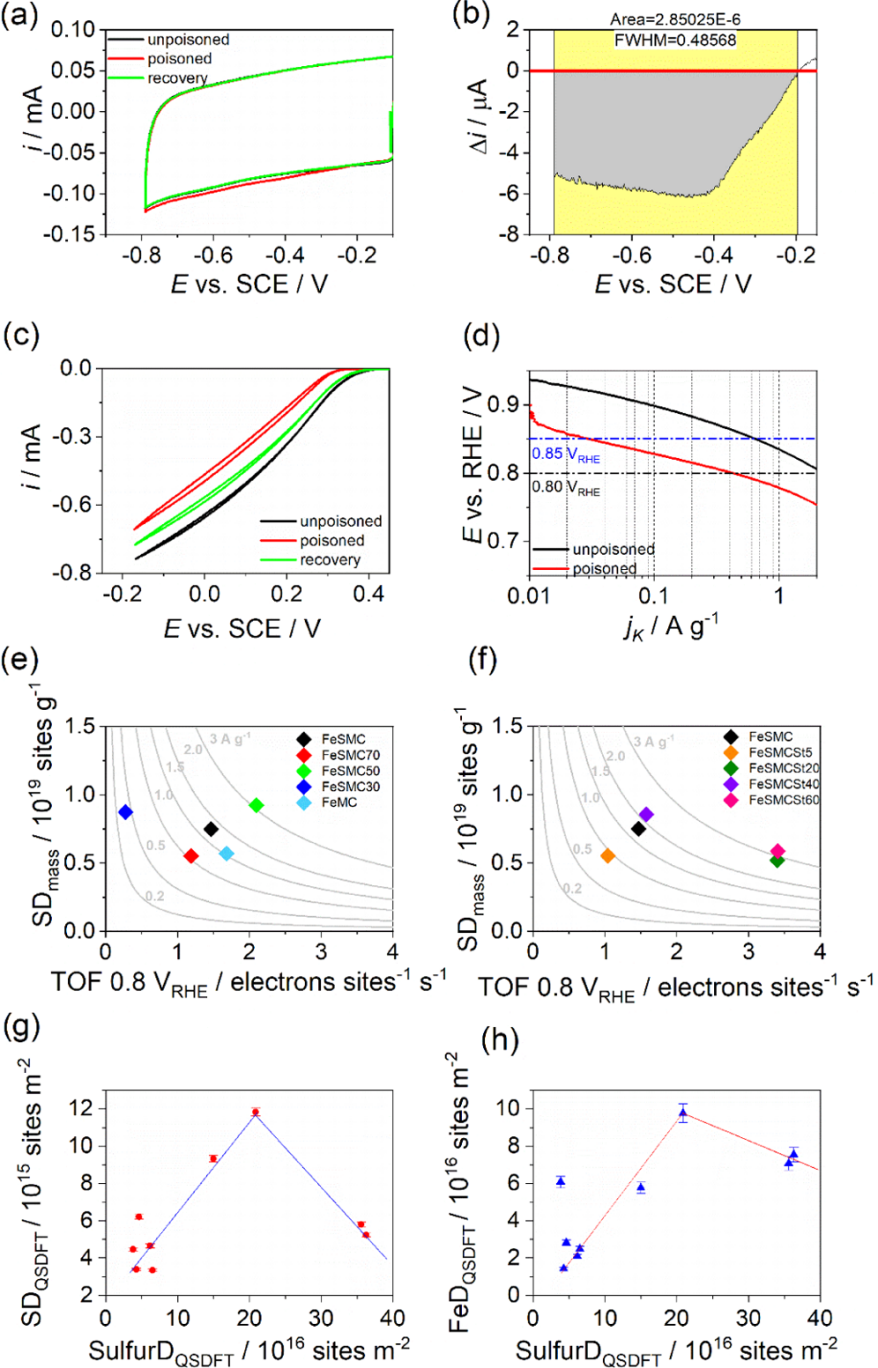
(a) Nitrite stripping and recovered CVs of FeSMC recorded in Ar-saturated electrolyte at 10 mV s^–1^, (b) magnification of the stripping area integrated for the site density determination, (c) LSV recorded before, during, and after poisoning at 1600 rpm and 5 mV s^–1^ in O_2_-saturated electrolyte, and (d) Tafel plots for the poisoned and recovered catalyst. Activity map of (e) FeSMCX series and (f) FeSMCStY series; gray lines represent the iso-current line, which is a hyperbole according to eq 5. (g) Variation of Fe–N*_x_* site density with respect to sulfur site density (SulfurD) in the catalysts. (h) Variation of Fe site density (FeD) with respect to sulfur site density in the catalysts. In (g) and (h), dotted lines are only intended to guide the eye.

To extend the electrochemical analysis and to compensate the uncertainty of XPS regarding the capability of identifying and quantify Fe–N*_x_* sites, we performed nitrite stripping to determine the SD and the turnover frequency (TOF) (Figure 6a,b).^41,74,75^ This procedure employs NO_2_^–^ as probe molecules able to reversibly poisoning Fe–N*_x_* sites by the formation of a NO–Fe bond. The electrochemical stripping of the nitrosyl ligand produces ammonia and the free Fe–N*_x_*.^75^ It is important to underline how, during the poisoning step, the activity is not totally suppressed because of the presence of other active sites such as nitrogen functional groups, which catalyze the different steps of the oxygen reduction reaction.^28^ Furthermore, SD determined by the NO method is 2 to 8 times lower than that derived from CO cryo-chemisorption, so it was proposed that SD_mass_ (NO_2_^–^) represents the lower bound of the SD_mass_ of Fe–N–C catalysts.

Figure 6c shows the activity toward the ORR of FeSMC at the different steps of the protocol: unpoisoned (black), poisoned by nitrite adsorption (red), and recovery (green) after nitrite reduction. The CVs in the potential range of nitrite reduction are reported in Figure 6a. The activity is almost recovered after nitrite stripping; in fact, the current profiles between −0.2 and −0.8 V vs SCE become superimposable. SD and TOF were calculated from the stripping charge obtained from the current difference between the CVs of poisoned and recovered samples according to eqs 4 and 5, respectively. The stripping current for FeSMC is plotted in Figure 6b, while Figure 6d compares the kinetic current density of unpoisoned and poisoned samples at 0.80 and 0.85 V vs RHE. This protocol was applied for all of the catalysts prepared, and the results are summarized in Table S4. FeSMC50 shows the highest SD (0.93 × 10^19^ sites g^–1^) of all of the investigated catalysts and the highest TOF (2.10 s^–1^) among the FeSMCX series. Among all sulfur-based catalysts, the highest TOF values are observed for the steam-activated samples, FeSMCSt20 and FeSMCSt60: TOF = 3.4 s^–1^ at 0.8 V vs RHE (Figure 6e,f and Table S4).

The reactivity maps reporting the SD variation versus the TOF are shown in Figure 6e,f. In these maps, each catalyst belongs to “iso-mass activity” hyperbolic curve, where the SD is normalized by the mass of catalysts [sites g^–1^]. The experimental data cannot be univocally interpreted on the basis of the sole sulfur content or textural properties, i.e., no clear correlation is individuated between SD or TOF and the sulfur content or the micropore surface. However, it can be observed that the catalysts prepared using a support with higher sulfur content show both high SD and TOF (FeSMC50 and FeSMC), whereas high TOFs are observed in those catalysts prepared using supports having high mesopore surface rather high sulfur content (FeSMCSt20 and FeSMCSt60). In fact, steam acts principally on mesoporosity, which is not involved in the formation of Fe–N*_x_* centers.^73^ Furthermore, the catalysts containing the highest values of SD (FeSMC50) does not coincide with the catalysts with the most positive *E*_1/2_ (FeSMCSt20) for the O_2_ reduction process. Therefore, this highlights that Fe–N*_x_* is important as active sites, but the activity depends also on other types of sites, which are more favorably utilized in those catalysts having high HF or micropore surface (Figure 5d,e). This is confirmed by the fact that iron percentage active for the ORR (utilization factor) increases in the steam-treated catalysts since the steam activation helps in opening up the structure, allowing a better access of electrolyte to the iron sites (Table S4).^23^ One last comment can be made considering Figure 6g,h. Figure 6g shows the plot between the Fe–N*_x_* site density and the “sulfur site density”, i.e., the number of sulfur functional groups (or sulfur atoms) per m^2^ of catalyst determined by N_2_ adsorption/desorption. The plot has a volcano-type shape where the highest number of active sites is determined in the catalyst (FeSMC50) having not too low or too high sulfur content, i.e., the optimal Sulfur/Fe–N*_x_* ratio (SulfurD/SD) is close to 18. As seen before, FeSMC50 has also a C–S–C/Fe ratio value very close to *r* = 1.8 determined by Ni et al. as to be the optimal one.^72^ However, it is worth mentioning that the sample having the best Sulfur/Fe–N*_x_* ratio is one of the most active, but not the most active at all because the HF is not optimal (Figure 5f). It is curious to observe that if instead of Fe–N*_x_* site density a more general Fe site density is considered, i.e., the number of iron atoms (determined by XPS) per m^2^ of catalyst, the trend has again a volcano shape, but the best ratio fairly matches with the 1.8 value determined by Ni et al.^72^ (Figure 6h). This is indicative that, in the best-case scenario, roughly 10% of iron sites are actively utilized for catalyzing the ORR. The different value between SulfurD/Fe–N*_x_* SD and SulfurD/FeD is strictly correlated to the utilization factor, i.e., not all of the available iron sites are accessible and involved in ORR catalysis and part of it is not even in the Fe–N*_x_* form. As further information, utilization factors calculated considering the Fe determined by ICP analysis are reported in Figure S4.

A last comment must be made about the possible electronic effect induced by the thiophenic functional groups on the Fe–N*_x_* sites, since the electron donation from thiophenic groups to the surrounding Fe–N*_x_* sites would modify the electronic structure of Fe–N*_x_*. Figure S2 reports the S 2p spectra for FeSMC, FeSMCSt20, FeSMC50, and the sulfur-doped carbon support SMC taken as reference. The Fe 2p_3/2_ XPS spectrum was of no help for the discussion since the very low concentration of iron does not allow a meaningful deconvolution. It should be noted that the S 2p peak of thiophenic sulfur in FeSMC50, which contain the highest Fe–N*_x_* sites, displayed a 0.2 eV positive shift compared with S 2p peak in SMC (Figure S2), and a value less than 0.1 eV is observed for FeSMC, which is the sample with the highest sulfur content. The observed BE shifts are too low to claim a sensitive electronic effect even if it cannot be excluded. However, these values must be taken with caution since they are of the same magnitude of the instrumentation sensitivity and the deconvolution loses its meaning when the sulfur content decreases far below 1% as in the case of FeSMC50. Therefore, we can conclude attesting that a suitable sulfur/iron ratio is important for the formation of Fe–N*_x_* active site; however, this does not directly translate the catalysts with the highest accessible Fe–N*_x_* SD in the most active catalysts, unless the textural properties are also optimized, i.e., there must be an optimized hierarchical porosity that allows better use of the active sites. Furthermore, even if we cannot exclude the presence of an electronic effect, we retain that other effects such as the hierarchical pore structures or the presence of a different content of Fe–N*_x_* active sites prevail for determining the final catalytic activity since the TOF, *j*_k_, and *E*_1/2_ do not change with increasing sulfur content.

## 4 Conclusions

In this work, we have simultaneously evaluated the effect of sulfur and textural properties on the formation of Fe–N*_*x*_*-type sites and, in general, on the catalytic activity of the prepared materials toward the oxygen reduction reaction. Five Fe–N–C catalysts were prepared from a carbonaceous support with different sulfur contents in the range 1–16 wt %. In other five catalysts, the effect of micro- and mesoporosities was evaluated by treating the carbonaceous support with steam at high temperatures so that the resulting Fe–N–C catalysts resulted in similar sulfur content but different textural properties (surface area varying from 1296 to 1726 m^2^ g^–1^). Fe–N–C catalysts were prepared by thermal treatment of mesoporous carbons with different sulfur content and Fe(Phen)_3_Cl_2_, which served as iron and nitrogen precursors. Crossing the data of EA, ICP, XPS, and N_2_ adsorption/desorption characterization with the electrochemical data for the oxygen reduction action and the SD determination, it was evaluated that there is no obvious dependence of the catalytic activity from the sulfur content, i.e., electronic effects induced by sulfur functional groups on iron sites appear to be not relevant. This was also confirmed by comparing the S 2p XPS spectra of different catalysts with the signal of the SMC carbon precursor. A very small shift (≤0.2 eV) to higher binding energy was observed, attesting that if an electronic effect is present, it is of small entity and is not expected to affect the catalytic activity of Fe–N*_x_* sites. On the contrary, the surface area of micropores and more generally on the HF factor, which defines an optimal ratio between area and surface of micro and mesopores, are more determinant for the electrocatalytic activity. It was found that the sulfur present in the synthesis of the carbonaceous material facilitates the formation of micro- and mesopores, and the sulfur remaining in the mesoporous carbon support facilitates the fixation of nitrogen functional groups, which like Fe–N_*x*_ centers are active or at least make active the nearby carbon atoms to the ORR. It has also been shown that the sulfur present in the carbonaceous support facilitates the formation of Fe–N*_x_* centers, but that this occurs for not too elevated sulfur content, otherwise FeS*_x_* formation results in an inevitable decrease in the Fe–N*_x_* yield. It is also observed that although sulfur facilitates the formation of active sites of type Fe–N*_x_*, this does not directly translate the catalysts with the highest Fe–N*_x_* SD in the most active catalysts. This is explained considering the presence of other active sites (pyridinic and pyrrolic) important in the ORR and for the necessary presence of a hierarchical porosity that allows a more effective exposure of Fe–N*_x_* and to improve the TOF of active sites. In summary, in this set of catalysts, sulfur participates indirectly in activity enhancement modifying the textural properties and/or the formation of active sites, while a direct electronic effect was not straightforward.

## Supporting Information Available

TEM images, S 2p XPS spectra, tables for Raman deconvolution parameters, element analysis by XPS of FeSMCx, electrochemical parameter from RRDE analysis, stripping data, and utilization factor (PDF)
