# Supplementary material for: Sulfur Doping versus Hierarchical Pore Structure: The Dominating Effect on the Fe–N–C Site Density, Activity, and Selectivity in Oxygen Reduction Reaction Electrocatalysis
Source: ACS Appl Mater Interfaces. 2021 Sep 1;13(36):42693–705. doi: 10.1021/acsami.1c09659 (PMC8447183; doi:10.1021/acsami.1c09659)
Supplement: Supplementary file 1 — am1c09659_si_001.pdf [file am1c09659_si_001.pdf]

Supporting Information

Sulfur Doping versus hierarchical pore structure: the dominating effect on the Fe-N-C site density, activity and selectivity in Oxygen Reduction Reaction electrocatalysis

*Giorgia Daniel, Marco Mazzucato, Riccardo Brandiele, Laura de Lazzari, Denis Badocco, Paolo Pastore, Tomasz Kosmala, Gaetano Granozzi, and Christian Durante**

Department of Chemical Sciences, University of Padova, via Marzolo 1, 35131, Padova, Italy.

S1 Supporting Figures pag. S2-S3

S2 Supporting Tables pag. S4-S5

S1 Supporting Figures

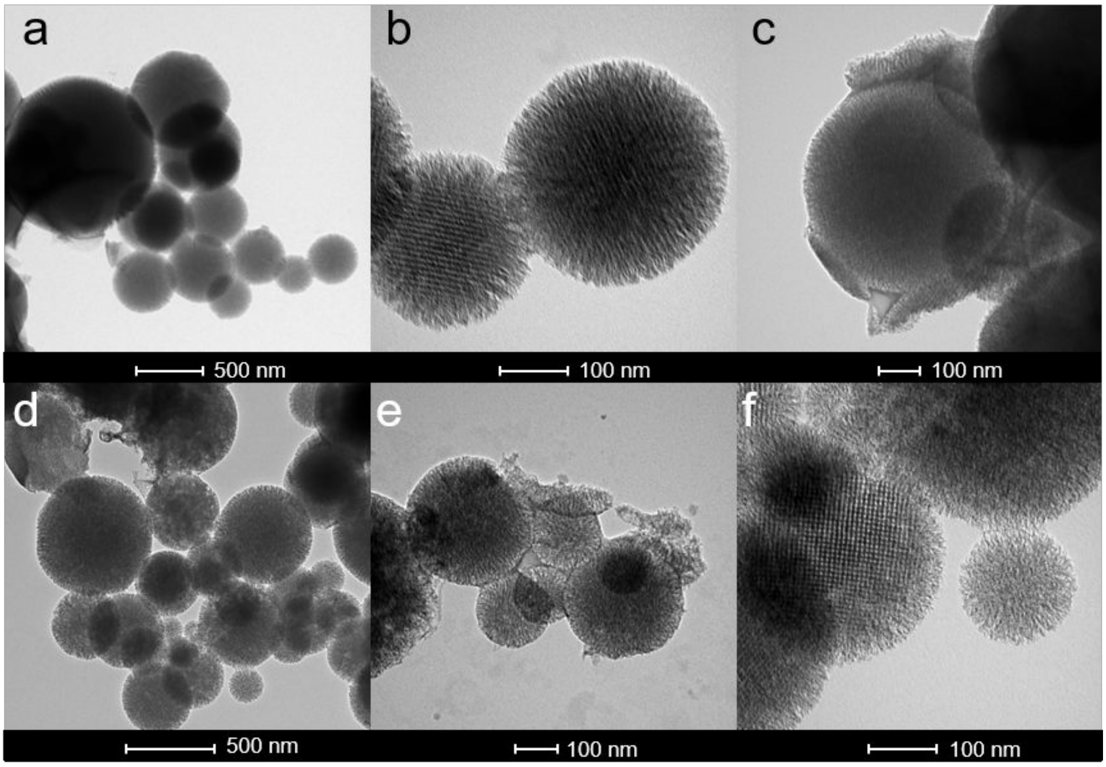

Figure S1: TEM image of (a-c) SMC100, (d-f) SMCSt60

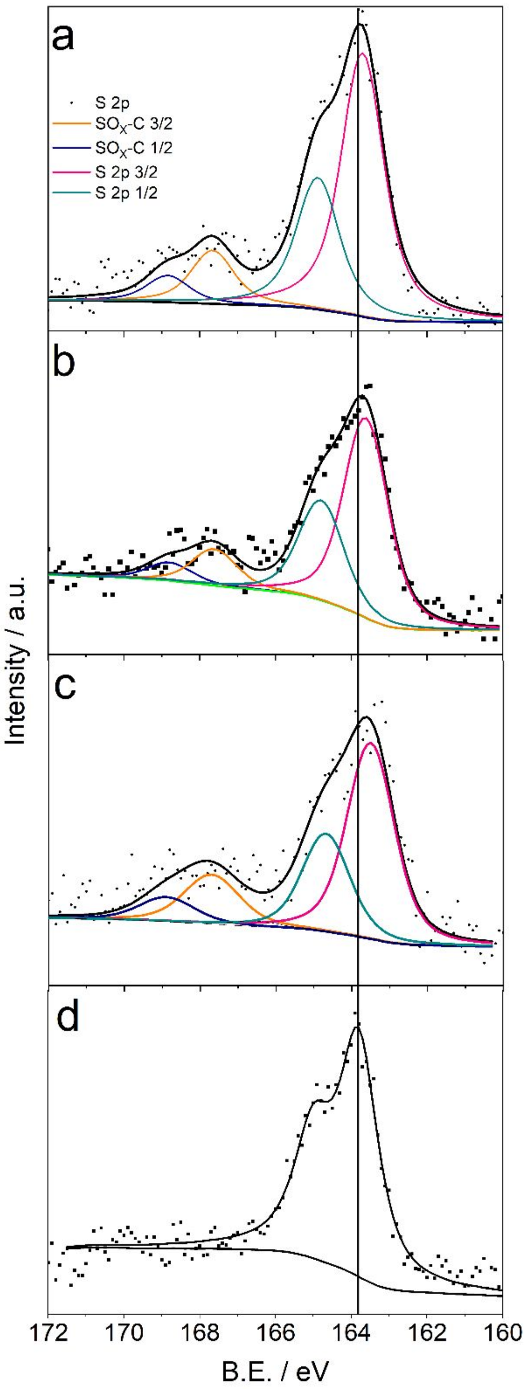

Figure S2: XPS spectra analysis of S 2p components of investigated catalysts (a) FeSMC, (b) FeSMC30, (d) FeSMC50, and (d) SMC.

S2 Supporting Tables

Table S1: Raman deconvolution parameter

	A_G cm^{-1}	A_{D3} cm^{-1}	D1 cm^{-1}	G cm^{-1}	I_{D1} a.u.	I_G a.u.	I_{D1}/I_G -	A_{D1}/A_G -	R_2 -
**FeSMC**	59.13	81.64	1333	1597	0.84	0.73	1.14	2.05	0.62
**FeSMC70**	53.57	104.30	1332	1599	0.76	0.69	1.11	2.08	0.55
**FeSMC50**	51.58	107.06	1328	1595	0.79	0.70	1.12	2.13	0.65
**FeSMC30**	61.33	104.78	1334	1600	0.83	0.76	1.10	1.96	0.65
**FeMC**	44.70	136.69	1328	1594	0.65	0.63	1.04	1.89	0.65
**FeSMCSt5**	53.25	79.38	1330	1596	0.82	0.74	1.11	2.10	0.57
**FeSMCSt20**	53.31	78.83	1330	1598	0.86	0.78	1.10	2.12	0.64
**FeSMCSt40**	54.12	80.84	1331	1597	0.89	0.77	1.15	2.21	0.62
**FeSMCSt60**	49.92	72.38	1327	1596	0.84	0.76	1.11	2.13	0.64

Table S2: Surface composition and differentiation derived from XPS analysis

	Fe wt.%	N wt.%	S wt.%	N_x %	N_{pyrid} %	N_{graf} %	N_{pyrro} %	C-S-C %	C-SO-C %	SOx %	SO_X/ C-S-C	r[a] -
**FeSMC**	0.85	1.36	2.45	14.5	23.7	12.7	32.1	79.3	1.8	18.9	0.24	4.0
**FeSMC70**	0.74	1.34	2.04	14.5	23.6	12.7	32.1	78.1	5.3	16.6	0.21	3.8
**FeSMC50**	0.53	1.06	0.79	19.9	20.3	11.8	31.3	73.7	4.2	22.1	0.30	1.9
**FeSMC30**	0.67	1.18	0.82	13.4	25.8	12.5	33.0	76.2	4.4	19.4	0.25	1.6
**FeMC**	0.72	1.02	0.26	15.7	19.9	13.6	31.6	64.9	0.0	35.1	0.54	0.4
**FeSMCSt5**	0.23	0.94	0.39	23.4	14.9	14.3	26.8	80.1	-	19.9	0.25	2.4
**FeSMCSt20**	0.36	0.80	0.54	20.3	20.4	11.8	31.8	82.9	5.8	11.3	0.14	2.2
**FeSMCSt40**	0.36	0.84	0.34	25.1	21.0	12.2	22.2	82.9	5.8	11.3	0.14	1.3
**FeSMCSt60**	0.23	0.65	0.39	13.3	26.6	8.7	29.3	65.1	-	34.9	0.54	2.0

[a] *C-S-C / Fe*

Table S3: Electrochemical parameter dervied from RRDE analysis at 1600 rpm 1 mV s^{-1} in 0.5M H_2SO_4

	$E_{1/2}$ V	j_l^{0V} mA cm^{-2}	$j^{0.8V}$ mA cm^{-2}	$j^{0.85V}$ mA cm^{-2}	$j_k^{0.8V}$ A g^{-1}	$j_k^{0.85V}$ A g^{-1}	*n* #	H_2O_2 %$^{0.7V}$	%$^{0.2V}$
**FeSMC**	0.725	4.37	0.660	0.162	1.30	0.28	3.97	3.5	1.9
**FeSMC70**	0.646	4.10	0.348	0.084	0.63	0.14	3.92	8.4	5.9
**FeSMC50**	0.695	4.94	0.660	0.182	1.27	0.31	3.91	2.7	1.2
**FeSMC30**	0.646	4.13	0.165	0.044	0.29	0.07	3.98	5.2	5.2
**FeMC**	0.717	4.35	0.561	0.127	1.07	0.22	3.93	4.8	3.1
**FeSMCSt5**	0.609	4.53	0.134	0.031	0.23	0.05	3.91	7.4	5.7
**FeSMCSt20**	0.739	4.76	0.840	0.193	1.70	0.34	3.95	2.6	4.1
**FeSMCSt40**	0.717	4.77	0.691	0.161	1.35	0.28	3.92	5.6	4.5
**FeSMCSt60**	0.731	4.97	0.660	0.147	1.27	0.25	3.93	4.4	4.3

Table S4: Stripping data and utilization factor *(Φ)* calculated from bulk Fe (ICP)

	SD	SD_{BET}	$TOF^{0.8V}$	$TOF^{0.85V}$	$SD_{MAX,ICP}$	***Φ***
	10^{18} sites g^{-1}	10^{15} sites m^{-2}	s^{-1}	s^{-1}	10^{19} sites g^{-1}	%
**FeSMC**	7.51	5.80	1.47	0.50	4.31	17.4
**FeSMC70**	5.54	5.24	1.19	0.33	7.33	7.6
**FeSMC50**	9.25	9.33	2.10	0.73	8.63	10.7
**FeSMC30**	8.75	11.8	0.28	-	11.8	7.4
**FeMC**	5.71	4.47	1.68	0.44	12.6	4.5
**FeSMCSt5**	5.54	4.65	1.04	0.34	2.70	20.5
**FeSMCSt20**	5.22	3.34	3.40	0.97	11.1	4.7
**FeSMCSt40**	8.56	6.22	1.58	0.38	11.1	7.7
**FeSMCSt60**	5.87	3.40	3.41	0.87	9.38	6.3
